# An alternative approach for complicated prosthetic aortic valve endocarditis

**DOI:** 10.1186/s40792-021-01195-7

**Published:** 2021-05-26

**Authors:** Dimos Karangelis, Argyris Krommydas, Fotios A. Mitropoulos

**Affiliations:** 1 Department of Cardiac Surgery, Democritus University of Thrace, University Hospital of Alexandroupolis, Alexandroupoli, Greece; 2 Department of Echocardiography Mitera Hospital, 15123 Athens, Greece; 3 Department of Cardiac Surgery, Mitera Hospital, 15123 Athens, Greece

**Keywords:** Prosthetic valve endocarditis, Sutureless valve, Aortic valve replacement, Pericardial patch, Patch exclusion technique

## Abstract

**Background:**

Surgical treatment of prosthetic valve endocarditis (PVE) with destruction of the aortic root and aortomitral continuity is demanding even in experienced hands.

**Case presentation:**

Herein, we describe a case of a 71-year-old female patient who presented with PVE that was further complicated by a fistulous abscess cavity. The patient underwent removal of the dehisced prosthetic valve, radical annular debridement, reconstruction of the aortomitral curtain with a pericardial patch as a patch exclusion technique and implantation of a sutureless valve.

**Conclusion:**

Patch exclusion technique, followed by sutureless valve implantation, might represent a feasible and safe alternative for the surgical treatment of complicated PVE.

## Background

Prosthetic valve endocarditis (PVE) is a serious complication which carries high mortality after valve replacement ranging from 20 to 80% [1]. Aortic annular erosion, abscess formation and destruction of the aortomitral continuity are markers of advanced disease. Here, we describe a case of sutureless aortic valve replacement (AVR) in a patient with PVE with destruction of the aortic root and spreading of the infectious process in the aortomitral curtain (AMC) with concomitant abscess formation and fistulization.

## Case presentation

A 71-year-old female patient was admitted under cardiology due to progressive dyspnea, NYHA Class III, flu-like symptoms and night sweats. From the past medical history, the patient was in end-stage renal failure under hemodialysis and was submitted to an AVR with a tissue valve (21 mm St Jude Trifecta) 3 years prior to the current admission. During work up, *Staphylococcus aureus* was isolated from the blood cultures and the patient was started on the daptomycin and rifampicin according to microbiology.

**Fig. 1:**
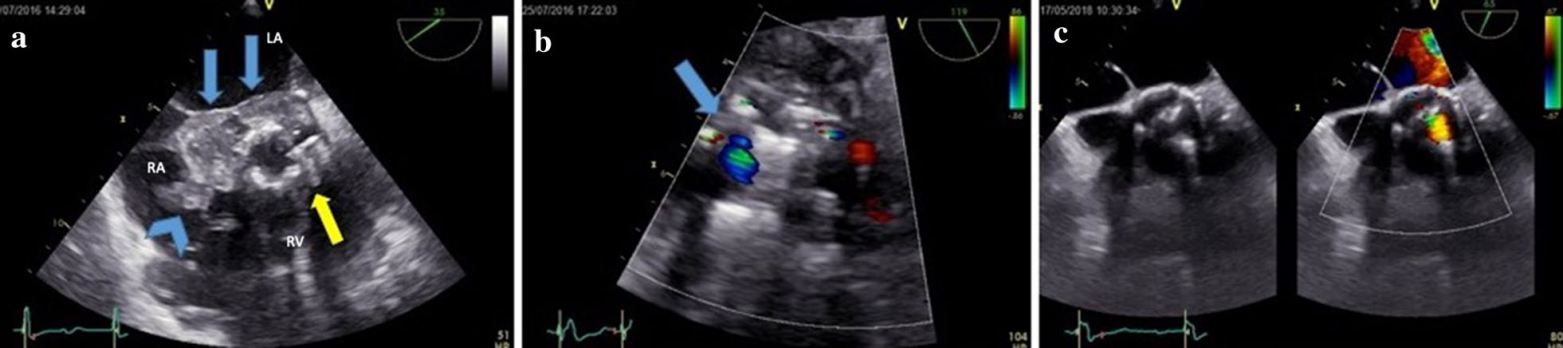
**a** Preoperative TEE short axis view. Blue arrows demarcate the large abscess cavity posteriorly. Blue chevron shows the vegetations in the right atrium. The severely calcified bioprosthesis is appreciated by the yellow arrow. RA: right atrium, LA: left atrium, RV: right ventricle. **b** Preoperative TEE long-axis view which shows the pseudoaneurysm formed by the ruptured abscess to the LVOT. **c** Postoperative TEE short axis view 2 years after surgery shows mild stenosis of the bioprosthetic aortic valve with no signs of abscess cavity or vegetations

Transesophageal echocardiography (TEE) revealed moderate stenosis of the previous prosthesis with paravalvular leak, preserved ejection fraction (EF) and the presence of an abscess cavity extending from the non- and the left coronary cusp to the roof of the left atrium. Furthermore, there were multiple vegetations in the right atrium (Fig. 1a, b) with a left ventricular outflow tract (LVOT)-to-right atrium fistula. A partial defect of the AMC reaching the anterior mitral valve leaflet associated with moderate mitral regurgitation was noted. The patient was referred for urgent surgery.

After redo sternotomy and arrest of the heart, transverse aortotomy revealed dehiscence of the aortic prosthesis along the non-coronary annulus and separation of the aortomitral continuity with abscess cavity formation and fistulization to the right atrium with presence of vegetations.

**Fig. 2:**
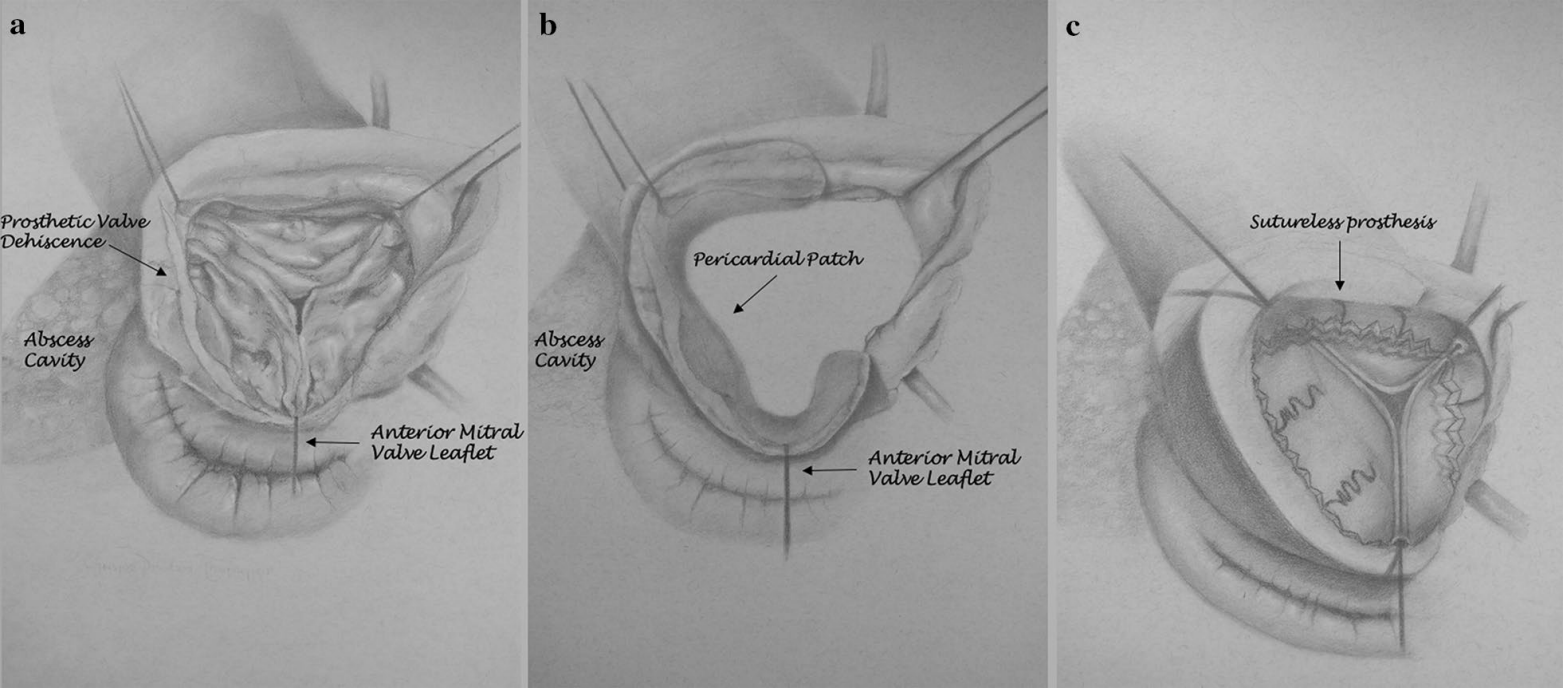
**a** Aortic prosthesis is dehisced along the non-coronary annulus and there is separation of the aortomitral continuity with abscess cavity formation. **b** Pericardial patch used for reconstruction of the large aortomitral continuity defect. **c** Deployment of sutureless valve

The abscess cavity was radically debrided and all friable tissue along with the prosthetic valve were removed. Pericardial patch was used for reconstruction of the large AMC defect. Posteriorly, the patch extended from the aortic wall adjacent to the left coronary ostium to the undersurface of the destructed aortic annulus. Inferiorly, it was sutured to the base of the anterior mitral valve leaflet and continued to the right cusp up to the point of the right coronary ostium. Superiorly, it was sutured to the aortic wall (Fig. 2a, b).

As there was barely any healthy tissue to suture a conventional prosthesis, we used a Perceval S® (LivaNova, Saluggia, Italy) size L sutureless valve (Fig. 2c) with our efforts focusing on seating the collar of the valve’s inflow above the annulus.

Subsequently, the right atrium was opened and the vegetations were removed. A small patch was also used on the atrial side to close the fistula and eliminate any communication with the left side and circulation in general.

The patient came off cardiopulmonary bypass easily and was transferred to the intensive care unit with minimum inotropic support. She had an otherwise uneventful postoperative course. Cultures of the excised prosthetic tissue came back negative.

On serial follow-ups, there was no recurrence of endocarditis and the abscess cavity has been resorbed. On a 2-year follow-up, echo showed mild-to-moderate stenosis of the prosthetic valve, trivial mitral regurgitation and preserved EF (Fig. 1c). No perivalvular leak was identified, while the mean and peak gradients were 16 mmHg and 28 mmHg, respectively. The effective orifice area (EOA) was 1.09, AV max was 2.62 m/s, the DVI 0.37, and the acceleration time was 85 ms. Moreover, LV ACC TIME/LV EJECTION TIME was 0.26.

## Discussion

In the context of such an extensive tissue destruction, the aortic annulus can be destroyed to such an extent that implantation of conventional prostheses becomes challenging. The early mortality after isolated valve replacement in complicated prosthetic valve endocarditis with conventional prostheses is reported to be as high as 9.7% with a 5- and 10-year survival of 87% and 75%, respectively [2].

In our case, we had no choice to implant a conventional prosthesis, as there was no annulus to support the valve sutures and the adjacent tissue was extremely friable. The rationale of covering the infected and destructed anatomic area with a patch was to achieve the following: (i) exclude this area from the circulation to minimize the risk of reinfection; (ii) reconstruct the anatomy; and (iii) provide a stable structural environment for the implantation of the new prosthetic valve.

In similar cases, full root replacement or even the commando operation has been proposed. These options, however, carry increased surgical risk. Use of homografts [2], or even allografts [3], has also been reported for similar pathology. Furthermore, an alternative approach would be the implantation of stentless bioprosthesis which minimize the burden of foreign material [4]. This approach, however, was deemed ineffective in our case, as there was no adequate tissue support.

Sutureless valves avoid the use of sutures in the annulus and allow the economy of precious cardiopulmonary and aortic cross-clamp time [5]. These valves rely on radial forces for stability and, therefore, provide efficient sealing of the annulus [6]. This technique has been utilized before with good results [1, 4, 7]. Rosello-Diez et al. report a mortality rate of 22.2% (2 patients out of 9) due to sepsis and multiorgan failure in the perioperative period [4]. Nevertheless, their results in short-term follow-up were satisfactory in terms of clinical and hemodynamic performance of the prosthesis with low reported transprosthetic gradients and mild periprosthetic regurgitation in one patient [4].

## Conclusions

PVE with extensive annular destruction and IVF disruption carries high morbidity and mortality and patients suffering from this clinical entity need early extensive surgery. Pericardial patch exclusion technique followed by sutureless valve implantation might represent a feasible and safe alternative for the surgical treatment of complicated PVE.

## Data Availability

Not applicable.

## Abbreviations

PVE: Prosthetic Valve Endocarditis
AVR: Aortic Valve Replacement
AMC: Aortomitral Curtain
EF: Ejection
LVOT: Left Ventricular Outflow Tract
EOA: Effective Orifice Area
